# MiMiR: a comprehensive solution for storage, annotation and exchange of microarray data

**DOI:** 10.1186/1471-2105-6-268

**Published:** 2005-11-09

**Authors:** Mahendra Navarange, Laurence Game, Derek Fowler, Vihar Wadekar, Helen Banks, Nicola Cooley, Fatimah Rahman, Justin Hinshelwood, Peter Broderick, Helen C Causton

**Affiliations:** 1 CSC-IC Microarray Centre, Imperial College, Hammersmith Campus, DuCane Road, London W12 ONN, UK

## Abstract

**Background:**

The generation of large amounts of microarray data presents challenges for data collection, annotation, exchange and analysis. Although there are now widely accepted formats, minimum standards for data content and ontologies for microarray data, only a few groups are using them together to build and populate large-scale databases. Structured environments for data management are crucial for making full use of these data.

**Description:**

The MiMiR database provides a comprehensive infrastructure for microarray data annotation, storage and exchange and is based on the MAGE format. MiMiR is MIAME-supportive, customised for use with data generated on the Affymetrix platform and includes a tool for data annotation using ontologies. Detailed information on the experiment, methods, reagents and signal intensity data can be captured in a systematic format. Reports screens permit the user to query the database, to view annotation on individual experiments and provide summary statistics. MiMiR has tools for automatic upload of the data from the microarray scanner and export to databases using MAGE-ML.

**Conclusion:**

MiMiR facilitates microarray data management, annotation and exchange, in line with international guidelines. The database is valuable for underpinning research activities and promotes a systematic approach to data handling. Copies of MiMiR are freely available to academic groups under licence.

## Background

Large amounts of microarray gene expression data are being generated by researchers attracted by the insights that can be gained from knowing the expression levels of thousands of genes [1]. In parallel, there has been a growing appreciation of the potential value of these data beyond the description found in most papers and for the need for well-annotated gene expression databases and mechanisms for data exchange [2]. Recognition of the role of standards in the management and use of microarray data has prompted a number of initiatives within the academic community [3,4]. The Microarray Gene Expression Data Society (MGED)[5], in particular, has made considerable progress towards providing guidelines and standards for the description, management and exchange of microarray data. MIAME, the Minimum Information About a Microarray Experiment, specifies the data content required to ensure that the data can be easily interpreted and the results independently verified [6]. MAGE provides a format for representation of all types of microarray gene expression data and includes both an object model (MAGE-OM) and a markup language (MAGE-ML) for data exchange [7]. The MGED Ontology supplies concepts, a controlled vocabulary and structure for describing the complete process of carrying out a microarray experiment [8-10]. Other groups within MGED are developing standards for data transformation, for other functional genomics technologies (the Reporting Structure for Biological Investigations Working Group) and are defining the Minimum Information Specification For In Situ Hybridization and Immunohistochemistry Experiments.

Support for these standards has come from multiple sources: manufacturers of hardware and software, the (US) Food and Drug Administration, which has endorsed MIAME as a standard for Voluntary Genomic Data submissions [11], and journals that have made the transfer of MIAME-compliant data into the public domain a precondition of publication [12-14]. There are now three public repositories for microarray data worldwide: ArrayExpress at the European Bioinformatics Institute [15-17], the Gene Expression Omnibus (GEO) at the National Cancer Institute [18,19] and the Center for Information Biology Gene Expression Database (CIBEX) at the DNA Data Bank of Japan [20,21].

Despite considerable advances in formats and standards for data content and description, and increased access to software and microarray data, few groups in academia are appropriately placed to take advantage of large-scale microarray data for high-throughput analysis. Some of the challenges lie in the generation of large data sets. Small perturbations in the microenvironment can have a large effect on the gene expression profile, so experimental manipulations need to be vigorously controlled and recorded [22]. The large number of interrelated variables make the data hard to annotate and only a few databases support the fine detail needed to make sense of these data [23,24]. As a result, many microarray facilities and academic groups lack resources for data management that support the existing standards and facilitate global data comparison and rigorous statistical analysis [2,25].

Here we describe MiMiR, an Oracle relational database, which was designed to assist users in tackling many of these challenges. This is a heavy-duty application used to collect, annotate, store and exchange microarray data and the related experimental information from thousands of arrays in a MAGE-ML compliant format. MiMiR has tools for data annotation that promote systematic use of the MGED and other ontologies, for importing data from the scanner and for exporting data to other MAGE-based databases. MiMiR has been optimised for fast and efficient data retrieval and high performance multi-user access.

## Construction and content

### Overview

MiMiR is a stand-alone application that was built to support work at an academic microarray centre. Staff at the Centre collaborate with researchers (users) to design experiments and to generate, manage, analyse, annotate and publish microarray data. MiMiR was therefore designed (i) to support flexible data collection and systematic annotation at a high level of detail, (ii) for data exchange with other MAGE-based databases and (iii) as the basis for a high-performance application for heavy-duty data analysis, mining and fast data retrieval by large numbers of concurrent users. These objectives have been achieved and the database is in constant use, although new functionality is continually being added via modular plug-ins.

### MiMiR architecture

**Figure 1:**
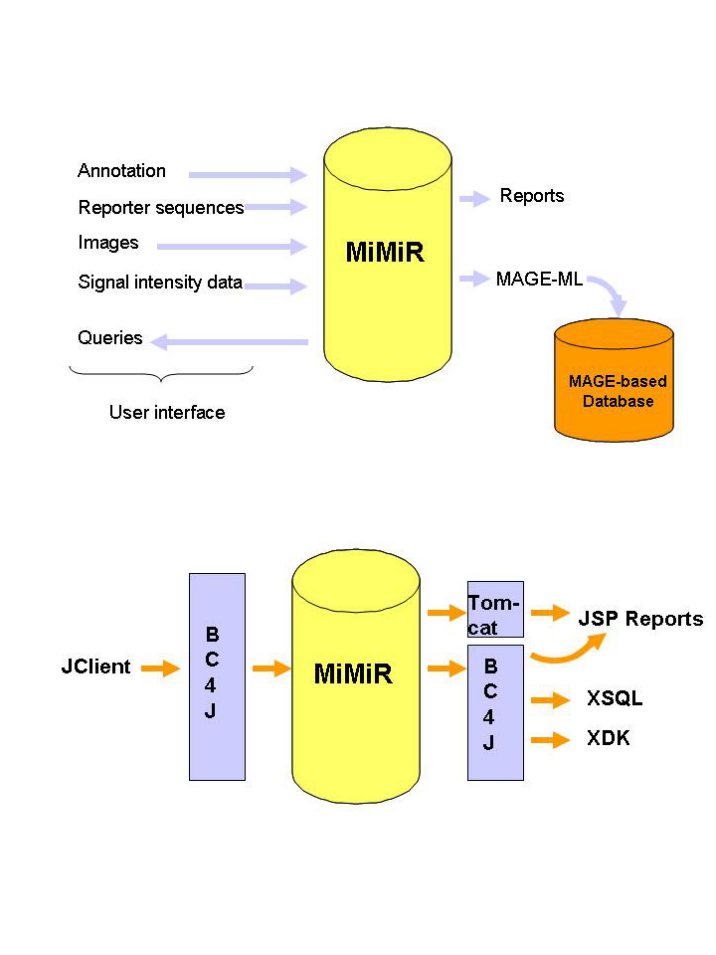
**Two views of MiMiR. **Figure 1a shows information flow in and out of the database. Information about the experimental design, spots on the array (the sequence of each reporter), signal intensities for each coordinate on the scanned array and the derived spot level data are uploaded into the database. The same interface is also used for queries and to update the data. Information about the data in MiMiR is available via web-based reports. Data on individual experiments may be exported as MAGE-ML to databases such as ArrayExpress. Figure 1b shows the architecture of the database and the underlying applications. The BC4J framework drives interactions between the user interface and the data model. JClient mediates navigation of the database. XDK permits the creation of XML directly from MiMiR. Tomcat offers potential for remote access of the database by users on different sites.

MiMiR is modular and consists of a fully integrated Oracle relational database, a custom-built Java user interface for data entry, management and the generation of reports, configuration software and a MAGE-ML export tool for data exchange with other MAGE-compliant databases (Figure 1a). The network database and web reporting application are server software, while the upload and export applications are client software.

Data stored in MiMiR include experimental annotation, the signal intensities for each coordinate on a scanned array, derived signal intensity data and the sequence present at each spot on the array. Data can be viewed using web-based reports, queried using the query interface and automatically exported as MAGE-ML.

MiMiR is a flexible application that was designed to support workflow at the Centre. The Centre operates on a service basis, generating data on large numbers of samples using a number of well-established quality control and standard operating procedures and primarily uses Affymetrix GeneChips. The staff annotate data on the biomaterial and treatments for researchers, frequently after hybridisation and scanning (information on the array design is obtained from the manufacturers and signal intensity data is loaded as part of the application). In consequence, the table structure and user interface were built to support flexible data capture, permitting data from multiple hybridisations to be loaded at once and linked to an experiment at a later stage. The structure was optimised for Affymetrix data and is built around standard operating protocols and quality controls, many of which are now used in other centres that generate microarray data using the GeneChip platform [26]. Information can easily be viewed, updated or modified using the same screens as those for data entry and there is provision for remote access.

The MAGE object model provides a comprehensive specification for representation of all types of microarray data and has more than 100 tables, many with only one or two columns [7]. Although data storage is well served by such a structure, the object model was not designed for efficient data retrieval, and many tables have to be traversed in order to access information. While remaining true to the data content requirements of MAGE-OM, MiMiR was built using a denormalised, purely relational, design in which columns and tables containing related information were combined. The design includes some deviations from the MAGE-OM: MiMiR permits multiple measurement units for each treatment in the Biomaterial Package, whereas MAGE-OM permits only one. Deviations such as this offer flexibility and save annotators time without compromising function. The table structure was designed so that many queries involve only the information in a single table. Querying is also facilitated by indexing the data using a few repeated primary keys that also link the tables. User-defined data types not supported by many simpler database platforms were avoided to enhance cross (database) platform interoperability. The use of a restricted number of array types with common design features meant that some of the tables in MAGE were not needed. The resulting table structure has only 35 tables.

Oracle-specific features were used to (a) improve data retrieval from large tables, (b) enhance data integrity and (c) obtain a modular application that is easy to maintain and navigate. Data in large tables were partitioned onto multiple disks and the partitions indexed such that data from the largest table containing raw signal intensities (37 GB) can be retrieved in less than 15 seconds (we use 4 disks). Data integrity is enhanced with constraints, sequences and indexes, e.g., triggers automatically check that references to data are in place upon data entry and that information uploaded, or modified, is updated in all the relevant records. SQL scripts regularly check for and report anomalies. Applications such as the BC4J framework provide tight integration between the user interface and the data model (Figure 1b).

### The user interface

#### Overview

A single interface is used for data entry, modifying records and querying the data. The screens are intuitive to use and are based around nine modules that represent the packages in MAGE. The modules provide a format for capturing information on all aspects of the experiment, from the researcher to the experimental design, arrays used, the biological sample, reagents (including catalogue numbers, suppliers and batch numbers), protocols, references, treatments, preparation of the labelled extract, hybridisation and scanning. Free text boxes provide space for recording additional information.

The interface was built using Oracle J Developer, which provides a sophisticated development environment tightly integrated with the database and application server [27]. The application is currently deployed as a .jar file and can be run on any PC that has JRE1.4.0.1, so that large numbers of individual users can access MiMiR from their desktops. The Business Components for Java (BC4J) framework (Oracle) is a tool for creating scalable high-performance J2EE applications and is implemented within JDeveloper. The software allows developers to build a logical persistence layer on which applications can be built and tested using Java objects. BC4J has two major advantages. Firstly, it mediates the interaction between the user interface and the data model (Figure 1b), freeing the developer from having to write, test and debug infrastructure code because applications only deal with the persistent data via Java objects. Secondly, it permits the creation of reusable objects and features 'Patterns' that encapsulate 'best practices' in J2EE coding e.g., Model-Viewer-Controller. The use of BC4J means that changes in the interface and database behaviour are easy to implement and modify and the database requires little maintenance.

JClient, used in the user interface, permits comprehensive navigation of MiMiR using predefined 'classes', or controls, while JSP and Tomcat provide potential for remote data retrieval. Data may be exported using XSQL and the GDAC Exporter [28]. Alternatively, XDK may be used to write XML directly from the database without the need for additional files. This makes XML-based data retrieval and export fast and efficient and requires minimal code.

**Figure 2:**
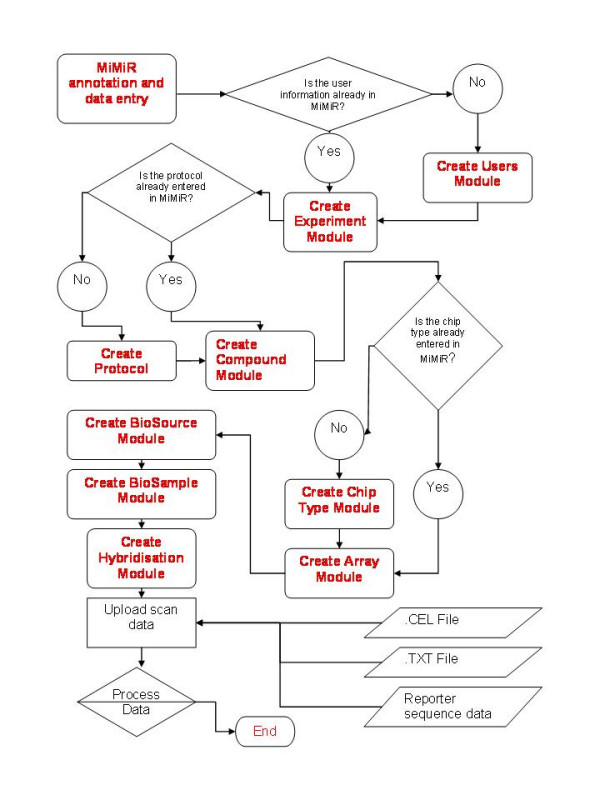
**Data captured in MiMiR. **Diagram showing the information captured from the details of the experiment through to the information gained from the scanned array and how they are linked. Although arrows indicate the logical flow of information capture as the experiment proceeds, data entry can be initiated at any of the boxes containing bold text and connected to other information about the experiment at a later stage.

The user interface mimics the logical flow of information to be recorded from the organism to the data generated from the scan (Figure 2). Data is typically generated before annotation, so MiMiR was built to be 'hybridisation-centric', as opposed to 'experiment-centric'. Annotation of experimental data can be initiated at any module and linked to the relevant scan at a later stage. Objects are tracked using unique identifiers, some of which are also used to link modules (e.g., the Array and Hybridisation modules are linked via the ChipID). Gene expression (scan) data are uploaded using an automatic function and labelled extracts are linked with the gene expression data in the Hybridisation module. Help is available in the form of pop-up windows for each field in the data entry application. There is also a comprehensive user manual [29] and a web-based annotation tutorial [30]. Quality control (QC) flags can be appended at a number of stages (total RNA, cRNA and scan) and are typically used for identifying problems in sample processing and to assist in data analysis.

#### Data annotation and MiMiR Ontology Viewer (MOV)

**Figure 3:**
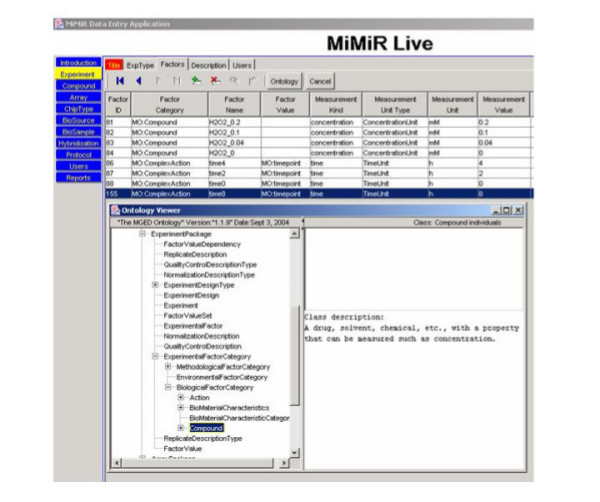
**A snapshot of the user interface showing how experimental factors are captured. **A snapshot of the data entry screen showing how the 'factors' (the variables) in an experiment are captured within MiMiR. The experiment involved treatment of cells in culture with 0, 0.04, 0.1 or 0.2 mM hydrogen peroxide and harvesting at 0, 2, 4 or 8 hours. The lower part of the screen shows the MiMiR Ontology Viewer (MOV) with the MGED Ontology displayed as a tree in the pane on the left. Definitions of terms highlighted in the tree are displayed on the right. The Factor Category and Value columns at the top of the screen were populated using MGED Ontology terms selected from MOV. The measurement kind, measurement unit type and measurement unit were selected from drop-down menus and the Factor Name and the Measurement Value were entered manually.

Annotation of experiments is important because the transcriptome is highly dynamic and changes rapidly in response to small perturbations [31]. However, detailed information about microarray experiments frequently remains in the laboratory notebooks of researchers and is not recorded in a form that is readily usable. Capturing this information is difficult because the variables that affect gene expression, and thus data interpretation, are not always known or accurately recorded. The use of ontologies can enhance the quality, accuracy and consistency of annotation, increasing the utility of the data for analysis and mining. However, there were no comprehensive ontologies, or controlled vocabularies, available until recently that could be used to describe this information systematically, and identifying the most appropriate terms and learning how to use ontologies can be challenging. The MiMiR data entry interface was therefore built with tools to encourage consistent annotation and the use of ontologies, to reduce problems associated with misspelling and different units and to minimise the use of free text. Where necessary, annotators can create their own terms and definitions. The MGED Ontology Viewer, or MOV, facilitates use of the MGED Ontology [8-10]. MOV displays the ontology as a dynamic class tree that can be searched and navigated (inset, Figure 3) and includes definitions for individuals. MOV is used to 'drill down' to locate terms in the ontology. Once the term has been identified and selected, the class and instance are automatically placed in the relevant fields in MiMiR, along with the 'MO:' designation that identifies the source of the term. Drop-down menus with predefined lists are used wherever possible. MOV can readily be used with any ontologies available in the DAML format and is based on SAX (Simple API for XML). Other ontologies, such as the NCI Metathesaurus [32] are also frequently used at the Microarray Centre. Examples of data annotation using MiMiR are available at [30].

The repeat function permits the data associated with an individual sample to be replicated. The application tracks the data across multiple tables and creates new records in each of the relevant tables respecting all primary and foreign key constraints. Individual fields can be modified to reflect minor differences among records. These functions ensure that a high level of detail can be captured, including samples that are pooled or split, with minimal typing and saves time when annotating large datasets.

#### Automated data upload

Expression data are loaded using an automated upload function and linked to the experimental information using a unique hybridisation identifier. Spot-level data typically load slowly, so data upload was enhanced using asynchronous processing. Files sent to MiMiR are automatically detected by the server, which creates six loader files, one for each of the tables storing spot-level data. The system administrators are emailed to inform them that upload occurred successfully or of the error logs to be checked.

#### Formats for viewing the data

A web reporting utility and reports screens provide summary information based on predefined queries, such as the amount of data in MiMiR and the types of arrays run. These are primarily used for checking data integrity.

#### Query tool

The query tool supports user-defined searches composed of single or multiple search criteria acting on single or multiple fields. The query result pages have 'clickable' screens that allow the user to navigate through records pulled out by a particular search.

#### Export of data as MAGE-ML

Export of data from MiMiR to other MAGE-based databases is carried out by converting the relevant data into MAGE-ML. Most of the data in MiMiR is sent to ArrayExpress upon publication and so an ArrayExpress export tool was developed. The export process involves retrieval of information about the experiment and the related biosources, biosamples, etc. using Oracle XSQL scripts. XML is generated directly from SQL statements run against the database. Affymetrix output files (.CEL, .CHP, and .EXP) are converted to MAGE-ML using the Affymetrix GDACExporter software [28]. The outputs from the XSQL and GDAC Exporter are merged using the eXtensible Stylesheet Language (XSL) to produce a single MAGE-ML file, validated and then transferred to the ArrayExpress ftp site.

## Utility and discussion

MiMiR provides a comprehensive data management environment and was built to support work at a busy academic microarray centre. A problem-based approach to design led to the creation of a purely relational database based on MAGE. MiMiR was built to capture and track information about experiments, as it becomes available, for exchange of data with other databases and provides the foundation for an application that supports higher level data analysis (Figure 1a). The database was designed as a high performance application and features a denormalised relational table structure, indexed primary keys that link the tables, data partitioned on multiple disks and an asynchronous data upload tool. Asynchronous data upload sets priorities and loads data when resources are available, which saves time and offers flexibility: an Affymetrix .CEL file of 15 MB typically loads in 10 seconds.

Most of the work carried out at the Microarray Centre is done on a service basis and the data entry interface was therefore built to support data upload via multiple access points (Figure 2). Information on each experiment is entered directly, at the level of detail chosen by the data annotator, and usually exceeds that required by MIAME. Details of researchers collaborating with the Centre and the title and description of the experiment are usually entered first; in other cases, the experimental data generated by hybridisation and scanning are entered first and the data annotated afterwards. Data can be updated using the same screens as those for data entry.

Figure 3 shows one of the 'Experiment' screens for a project, carried out in collaboration with the Centre [33]. Hydrogen peroxide at 0, 0.04, 0.1 or 0.2mM was added to rat neonatal myocytes in culture and cells were harvested at 0, 2, 4 or 8 hours after treatment. The 'factors', or intended experimental variables, are the 'compound' hydrogen peroxide and the 'timepoints' at which the samples were harvested. MGED Ontology terms have the 'MO:' prefix. Terms of the predefined 'Measurement Kind', 'Measurement Unit Type' and 'Measurement Unit', were chosen from dropdown menus. MOV was used to select the Factor Category and Values from the BiologicalFactorCategory class from a hierarchical view of the ontology. The terms highlighted by the annotator are automatically pasted into the relevant fields in the data entry screen. Here, text was only entered manually in the Factor Name and Measurement Value columns.

Although the treatment of the cells with hydrogen peroxide is unique to this experiment, many of the subsequent stages, in which total RNA was prepared and the samples were labelled, fragmented, hybridised to arrays and scanned, differ little among experiments and are carried out using standard operating protocols [26]. In consequence, data entries for the latter stages of sample preparation were linked to the commonly used protocols. The data were annotated using fields for recording sample and experiment-specific details such as batch numbers of reagents, deviations in protocols, settings, yields and quality control information. The experimental information for one sample was completed, the whole record was duplicated using the repeat function and then modified for subsequent samples.

The data was submitted to ArrayExpress using the export tool and can be accessed from [34] (E-MIMR-12). Although the database is currently only used for MAGE export, MAGE import can be carried out using Oracle's XQuery tool [35]. A parser is only needed for situations where the MAGE imported is systematically different from that generated in house, for example, where information stored in a field in MiMiR is coded as free text.

### Comparison with other systems

Many groups in the microarray data generating community recognise the importance of large scale databases for data exchange, annotation and management and there are now three public repositories of microarray data all of which accept gene expression data from multiple platforms. GEO uses the Simple Omnibus Format in Text format, while ArrayExpress and CIBEX use MAGE for data exchange. Some of the larger databases that support academic communities are the Stanford Microarray Database [36-38] that holds array data from the spotted array platform and the RNA Abundance Database [24,39,40] that holds data from multiple platforms.

MiMiR shares similarities to RAD in that both support discrete academic communities generating microarray data, are based on MAGE, and have been built for efficient querying and data retrieval, both provide extensive tools for data annotation and the use of ontologies and support export of data to ArrayExpress. BASE and MARS are two other comprehensive microarray management systems [41,42]. Like MiMiR they are also designed to support large numbers of users and to capture information usually found in a LIMS. Unlike MiMiR, they permit the user to carry out data transformation and to store the intermediate results of analysis, however, they have less extensive tools for data annotation and the systematic use of ontologies. BASE accepts Affymetrix, two colour and comparative genome hybridisation arrays, while MARS is built for multicolour arrays only. MiMiR also shares features with The Public Expression Profiling Resource (PEPR) [43]. Both of these Oracle databases are built around the Affymetrix platform and support data generation at microarray centres.

### Future work

Planned additions to MiMiR include extension of the object model to accommodate data from multiple applications of spotted arrays and the annotation associated with clinical samples. Development of a web-based portal integrating multiple data analysis tools and integration of the database with Rosetta Resolver [44] are currently underway.

## Conclusion

MiMiR is an Oracle relational database for microarray data storage, annotation, export and analysis that supports MAGE and MIAME compliance. The application is used for data management across a large and dispersed user-base and draws heavily on MGED standards and data structures.

Advantages of using MiMiR include:

• Data structure optimised for fast and accurate data retrieval, supporting large numbers of users (our implementation contains data from more than 2000 hybridisations (more than 70 GB)).

• Flexible user interface that permits data entry as data accumulates.

• Extensive focus on accurate and consistent sample annotation and data tracking. Including features for recording detailed experimental information, viewing and using ontologies, repeating records, generating web-based reports and querying the data.

Despite differences in the microarray gene expression databases and platforms available, support for common standards, the ability to share data and the willingness of investigators to put their data into the public domain are enhancing the utility of all data. This, in turn, is yielding greater insights into the biological systems underlying these data and predicts a strong future for knowledge gained from use of array-based technologies.

## Availability and requirements

Project Name: MiMiR

Project Homepage: 

Server Operating System: MS Windows (2000/XP/Server2003). MiMiR has a modular design and has been developed using standard tools such it can be modified to run under other operating systems and database environments. However, we do not anticipate that the entire system can be ported as a unit because features such as the Perl upload services, asynchronous data processing, GDAC Exporter and BC4J are platform-specific.

Client (User Interface) Operating System: All 32-bit MS Windows (95/98/NT/2000/XP)

Database Requirements: Oracle 9i

Programming Language: Java, Perl.

Copies of MiMiR are freely available to academic groups under license. Further information and requirements using the database management system can be obtained from [29].

## Authors' contributions

MN and HC designed and conceived of the database. MN carried out the high level design, developed and implemented MiMiR. LG worked on comprehensive data annotation, management and coordination of the later stages of the project. MN, LG and HC drafted the manuscript. DF developed the user interface, the web reports and worked to enhance the Ontology Viewer. LG, HB, NC, FR and PB generated and annotated microarray data and populated and tested the database. VW developed the first versions of the Ontology Viewer. JH developed the ArrayExpress export tool. HC guided and coordinated execution of the project. All authors participated in optimising the design of MiMiR and have read and approved the final manuscript.
